# The protein conformational basis of isoflavone biosynthesis

**DOI:** 10.1038/s42003-022-04222-x

**Published:** 2022-11-15

**Authors:** Xiaoqiang Wang, Haiyun Pan, Someswar Sagurthi, Vincent Paris, Chunliu Zhuo, Richard A. Dixon

**Affiliations:** 1 BioDiscovery Institute and Department of Biological Sciences, University of North Texas, Denton, TX 76203-5017 USA; 2 Conagen, Inc., Bedford, MA 01730 USA; 3 Department of Genetics & Biotechnology, Osmania University, Hyderabad, 500007 India

**Keywords:** X-ray crystallography, Secondary metabolism

## Abstract

Isoflavonoids play important roles in plant defense and also exhibit a range of mammalian health-promoting activities. Their biosynthesis is initiated by two enzymes with unusual catalytic activities; 2-hydroxyisoflavanone synthase (2-HIS), a membrane-bound cytochrome P450 catalyzing a coupled aryl-ring migration and hydroxylation, and 2-hydroxyisoflavanone dehydratase (2-HID), a member of a large carboxylesterase family that paradoxically catalyzes dehydration of 2-hydroxyisoflavanones to isoflavone. Here we report the crystal structures of 2-HIS from Medicago truncatula and 2-HID from Pueraria lobata. The 2-HIS structure reveals a unique cytochrome P450 conformation and heme and substrate binding mode that facilitate the coupled aryl-ring migration and hydroxylation reactions. The 2-HID structure reveals the active site architecture and putative catalytic residues for the dual dehydratase and carboxylesterase activities. Mutagenesis studies revealed key residues involved in substrate binding and specificity. Understanding the structural basis of isoflavone biosynthesis will facilitate the engineering of new bioactive isoflavonoids.

## Abstract (web summary)

The structure and function of two isoflavone biosynthetic enzymes are reported revealing a novel cytochrome P450 conformation and identification of key residues for dual dehydratase and carboxylesterase activities

## Introduction

**Fig. 1:**
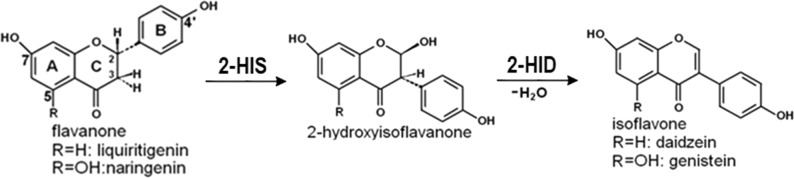
The enzymatic reactions catalyzed by 2-HIS and 2-HID. 2-HIS catalyzes hydroxylation and aryl-ring migration of flavanones (e.g., liquiritigenin and naringenin) to convert them to 2-hydroxyisoflavanones, and 2-HID then catalyzes dehydration of 2-hydroxyisoflavanones to yield the final isoflavone products, e.g., daidzein or genistein.

The isoflavonoids occur primarily in legumes and play important roles in plant defense. They also have various estrogenic, antiangiogenic, antioxidant, and anticancer activities, leading to their popularity as dietary supplements^1–4^. The isoflavone genistein can inhibit the growth of cancer cells through modulation of genes that are related to the control of cell cycle, apoptosis, and cell signaling pathways^5,6^. Isoflavonoids are synthesized through the central phenylpropanoid pathway and the specific isoflavonoid branch pathway. 2-Hydroxyisoflavanone synthase (2-HIS) catalyzes the entry-point reaction into isoflavonoid biosynthesis to convert flavanone to (2R, 3S)-2-hydroxyisoflavanone^1,7^. 2-Hydroxyisoflavanone dehydratase (2-HID) then catalyzes dehydration of 2-hydroxyisoflavanones to yield the first isoflavone products, daidzein or genistein^8^ (Fig. 1). *2-HIS* genes have been characterized from leguminous plants including licorice (*Glycyrrhiza echinata*)^9^, soybean (*Glycine max*)^10^, and the model legume *Medicago truncatula*^11^. 2-HID has been identified and characterized in soybean^8^, licorice (*Glycyrrhiza echinate)*^8^, kudzu (*Pueraria lobata*)^12^, and *Lotus japonicus*^13^.

2-HIS is a membrane-associated cytochrome P450 enzyme belonging to the CYP93C subfamily. It catalyzes a unique reaction involving both aryl-ring migration of the aromatic B-ring from position C2 to C3 and a coupled hydroxylation reaction adding an oxygen atom at position C2^9^. 2-HID is classified into a large carboxylesterase family, but may not function for ester hydrolysis in vivo, although some weak carboxylesterase activity has been demonstrated in vitro for soybean 2-HID with *p*-nitrophenyl butyrate^8^. 2-HID functions in vivo as a dehydratase for dehydration of 2-hydroxyisoflavones. The molecular bases for the aryl-ring migration and hydroxylation functions of 2-HIS and the hydrolysis and dehydration functions 2-HID are not understood, and neither are their catalytic mechanisms and substrate specificities.

A very large number of P450s are present in plants, many of which are involved in the biosynthesis of natural products^14,15^ and catalyze oxidation of diverse substrates in primary and secondary metabolism^16^. Currently, only a few crystal structures are available for this very large and important class of plant enzymes, including two class III peroxide-metabolizing P450 allene oxide synthases^17,18^, and several plant P450s with only hydroxylation activity, e.g., cinnamate 4-hydroxylase (CYP73A33) from *Sorghum bicolor*^19^, CYP76AH1 from *Salvia miltiorrhiza*^20^, and CYP90B1, CYP97A3 and CYP97C1 from *Arabidopsis thaliana*^21,22^. The mechanisms of more complex plant P450-mediated reactions such as aryl-ring migration are not yet understood.

Here we report the structures of 2-HIS from *M. truncatula* and 2-HID from *P. lobata*. The 2-HIS crystal structure reveals new heme and substrate binding modes in a different conformation from all P450s with known structures and provides a basis for understanding new reaction mechanisms and substrate- and regio-specificities within the plant P450 superfamily. The 2-HID structure reveals features different from other known dehydratase structures but similar to carboxylesterases. A comparative structural study further identified the putative binding sites for the substrates, the active site architectures and the catalytic residues for the dehydratase activity and carboxylesterase functions. Mutagenesis studies of both enzymes revealed key residues involved in substrate binding and specificity.

## Results and discussion

### 2-HIS Structure

**Fig. 2:**
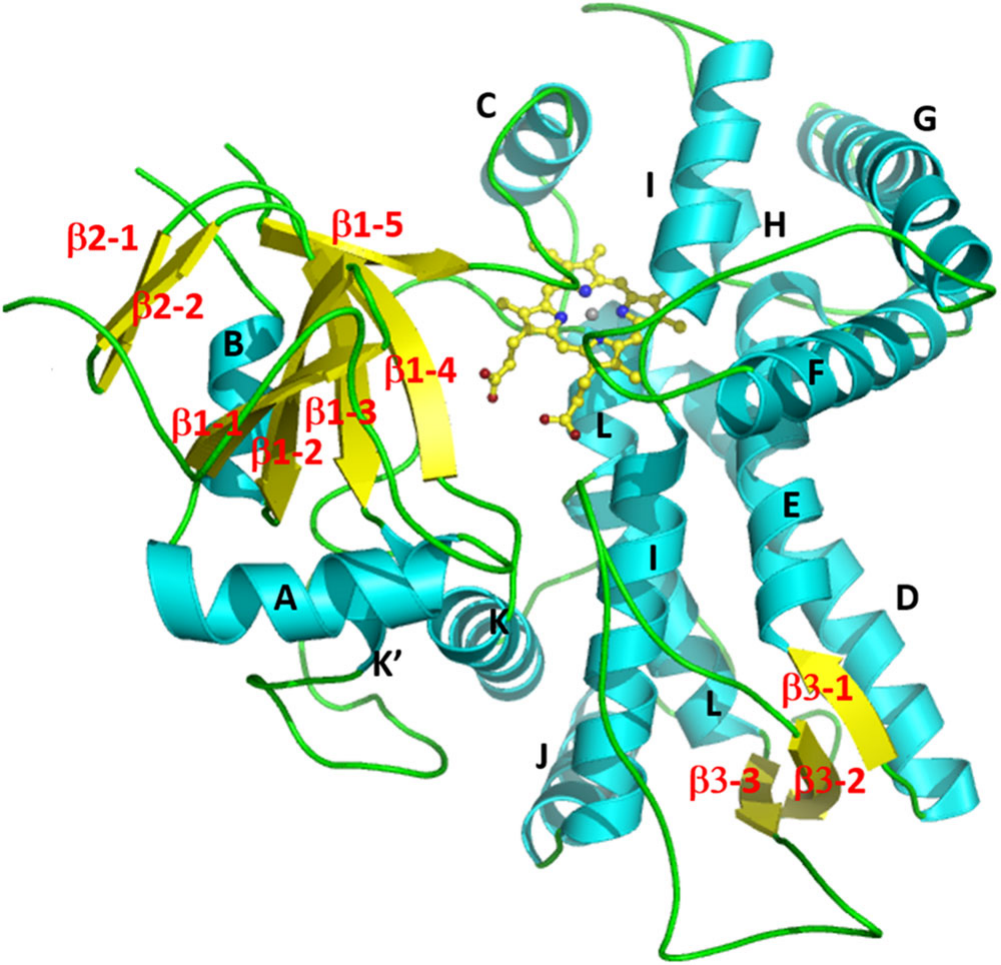
Ribbon diagram of the M. truncatula 2-HIS structure. The α helices, β strands and the N- and C-termini are labeled. Fig. 1–6 were prepared with MOLSCRIPT^51^ and RASTER3D^52^, or PyMOL^53^.

The crystal structure of *M. truncatula* 2-HIS was determined using the multiwavelength anomalous dispersion method with a selenomethionine-substituted enzyme crystal in a *P*2_1_2_1_2_1_ space group. Another 2-HIS native crystal form with a *P*2_1_ space group diffracted to 2.0 Å resolution, and the structure was determined using the molecular replacement method (Table 1). 2-HIS exhibits a common CYP fold^23^ and contains two domains with three β sheets and thirteen α helices (Fig. 2, Supplementary Figs. 1, 2). There are two molecules in an asymmetric unit which form a dimer (Supplementary Fig. 3) with extensive interactions. The structures of two molecules in the asymmetric unit are highly similar to each other with a root mean square deviation (RMSD) of 0.59 Å for 451 Cα atoms. The overall structure of 2-HIS is also similar to that of other P450s, including human P450 2C9 with an RMSD of 2.52 Å for 239 Cα atoms (Supplementary Figs. 1, 2).

**Table 1:** Summary of 2-HIS data collection and refinement statistics.

Data statistics	Native	Se peak	Se inflection
Space group	*P*2_1_	*P*2_1_2_1_2_1_	*P*2_1_2_1_2_1_
Unit Cell	a = 50.1 Å	a = 73.8 Å	a = 73.9 Å
	b = 73.8 Å	b = 96.0 Å	b = 96.0 Å
	c = 148.7 Å	c = 154.6 Å	c = 154.6 Å
	β = 93.5°		
Resolution(Å)	2.0	3.0	3.0
Wavelength(Å)	0.97899	0.97905	0.97918
Unique reflections	71557 (6400)	22817 (2496)	22829 (2243)
Completeness(%)	98.0 (87.78)	99.9 (99.8)	99.9 (99.8)
R_sym_ (%)*	6.9 (36.6)	18.1 (68.9)	18.4 (76.9)
I/σ(I)	17.9 (2.2)	10.2 (3.2)	10.5 (2.7)
Figure of merit		0.44	
*Refinement Statistics*			
R factor (%)	19.16		
R_free_ (%)	22.78		
Number of protein atoms	7154		
Number of water molecules	463		
Average B-factors (Å2)	44.0		
R.m.s.d. from ideal values			
Bond length (Å)	0.003		
Bond angle (°)	0.525		
Ramachandran plot			
Favored (%)	97		
Allowed (%)	3		
Outliers (%)	0		

^*^R_sym_ = Σ_hkl_ | *I*- < *I* > | /Σ*I*, where *I* is the observed intensity and <*I* > is the average intensity from observations of symmetry-related reflections. A subset of the data (10%) was excluded from the refinement and used to calculate the free R value (R_free_). R factor = Σ||F_o_ | -|F_c_ | |/Σ|F_o_ | . Numbers in parentheses are for the highest resolution shell.

### 2-HIS heme binding mode

**Fig. 3:**
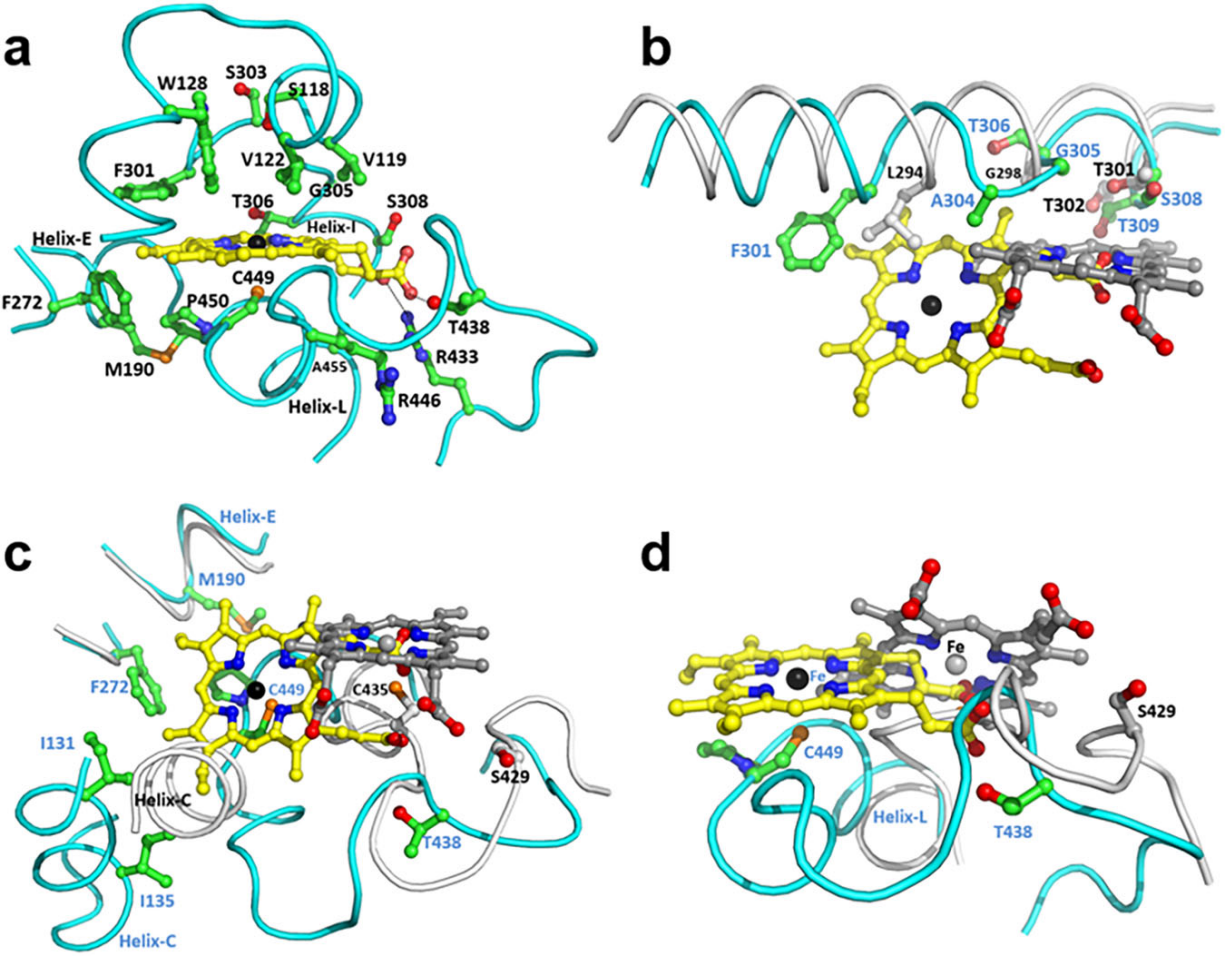
The heme binding conformation of 2-HIS. **a** Heme molecule and its interactions with 2-HIS. **b** A comparison of the I-helices of 2-HIS (in cyan) and human P450 2C9 (in grey). **c**,**d** A comparison of the heme binding loop of 2-HIS (in cyan) and human P450 2C9 (in grey). The structures of heme and key residues involved in heme binding are shown as ball-and-stick models and colored according to element: oxygen, red; nitrogen, blue; sulfur, orange; iron, black for 2-HIS, and grey for P450 2C9; and carbon, green and yellow for 2-HIS residues and heme, and grey and dark grey for P450 2C9 residues and heme. The same atom color codes are used for other figures except ones with specific descriptions. The dashed lines in Fig. 2a indicate the interactions between heme and R433 and T438.

The heme prosthetic group was observed in the structure of 2-HIS with well-defined electron density (Supplementary Fig. 4) and is located mainly between helices I and L with the I-helix on the distal side and L-helix on the proximal side, surrounded by helices C and E and the loop before helix C (Fig. 3a–d). Cysteine 449 acts as the 5th ligand for the iron of the heme cofactor. Remarkably, however, the location and orientation of the heme are quite different in 2-HIS compared with other P450s (e.g., human P450 2C9). The heme is moved, relative to that in human P450 2C9^24,25^, toward helices C and E, with a distance of ~8 Å between the heme iron in the two structures, and helix C has also shifted, by ~7-8 Å (Fig. 3c). Compared with P450 2C9, the plane of the heme protoporphyrin ring in 2-HIS is tilted down ~50° (Fig. 3b). The two propionate groups also rotate ~90° towards the substrate binding pocket, a very different configuration from classical P450s, in which both propionate groups form a strong charge-charge interaction with Arg or other basic amino acids. In the structure of 2-HIS, Arg433 and Arg446 are close to the heme pyrrole rings C and D. This region is flexible in the 2-HIS structure, with different conformations in molecules A and B in the asymmetric unit. Arg433 in molecule B forms a charge-charge interaction with the propionate group on the heme pyrrole ring D, but Arg433 in molecule A is relatively far away from the heme, indicating that the interaction between Arg433 and heme is dynamic. This location is near the substrate binding site as described below, and the dynamic interaction and conformation are likely important for the substrate binding and catalysis.

The classic P450 monooxygenases contain a signature sequence (A/G-G-x-D/E-T-T/S) in the I-helix^26^, regarded as an oxygen binding motif with the conserved glycine (Gly298 in human P450 2C9) pointing at the heme and the conserved threonine (Thr301 in P450 2C9) pointing to the oxygen binding site. The corresponding sequence is conserved in 2-HIS, although Ser308 substitutes for the corresponding threonine. However, the conformation of the I-helix and its interactions with the heme in 2-HIS are different from those in P450 2C9 (Fig. 3c). Since the heme group shifts towards the N-terminus of the I-helix, Gly305 and Ser308 in 2-HIS are far away from the heme iron and the “oxygen binding site”. Ser308 still interacts with the heme at a distance of ~3.6 Å to a propionate group of the pyrrole ring D. Ser308 also points to the putative substrate binding pocket and could interact with substrate (Fig. 3b).

The I-helix in 2-HIS is totally distorted in the middle (S303-T306) region which is elongated without a classic helix pattern, and also shifts ~2-3 residues towards the N-terminal end of the I-helix in comparison with P450 2C9 (Fig. 3b). The heme-binding loop, Thr438-P450, is located on the proximal face of the heme just before the L-helix (Fig. 3c, d). The conformation of the N-terminal region of the heme-binding loop of 2-HIS is quite different from that of other P450s; the loop is longer and more extended in the N-terminal end with Thr438 forming a hydrogen bond to a propionate group of heme pyrrole ring C, accommodating the heme movement of ~8 Å. The middle and N-terminal portions are extended towards the heme protoporphyrin ring since the ~90° rotation of the heme with its propionate group frees space for this new conformation.

### 2-HIS oxygen- and substrate-binding sites

In most P450s, the oxygen and substrate binding sites are on the distal side of the heme. In the 2-HIS structure, electron density was observed on the top of the heme iron, which may be fitted by a water molecule. The oxygen binding pocket is relatively small, surrounded by amino acids Ser118, Val119, Val122, Trp128, Phe301, Ala304, and Thr306 (Fig. 3a, b). This pocket could also bind oxygen but is not big enough to serve as the substrate binding pocket. Trp128 and Phe301 could be important for controlling access and binding of oxygen.

**Fig. 4:**
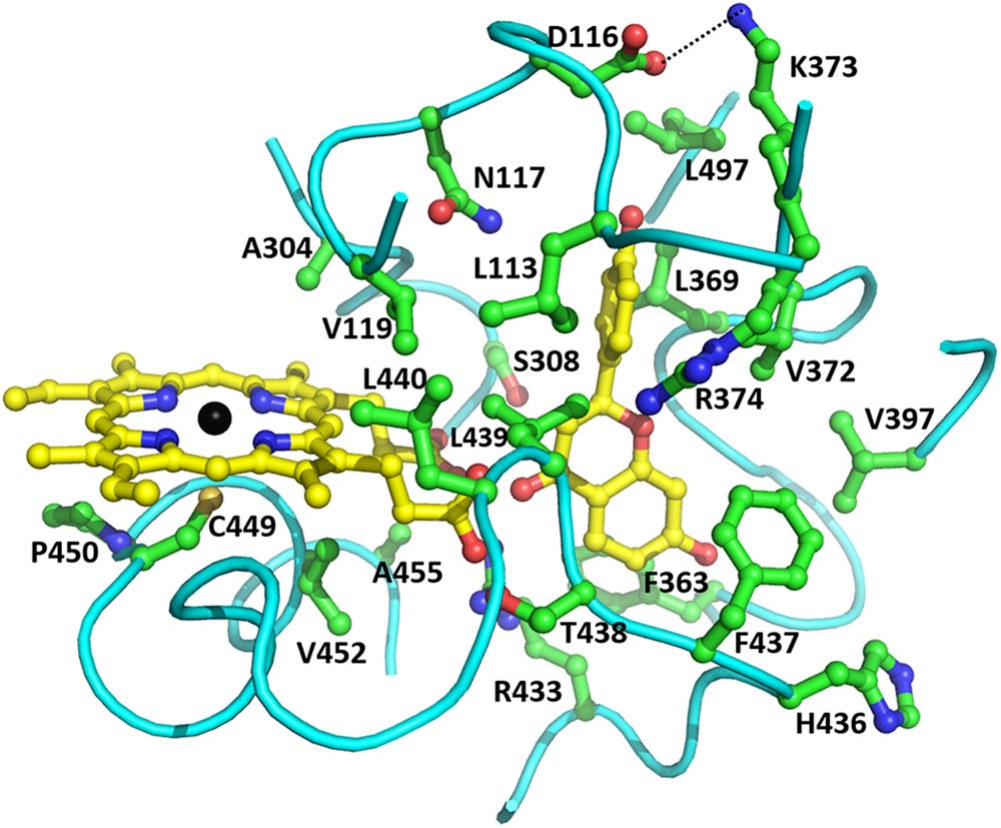
The substrate-binding pocket and interactions of 2-HIS with docked substrate liquiritigenin. Substrate liquiritigenin and heme are shown as ball-and-stick models with carbon atoms colored in yellow and iron atom in black. Some amino acid residues in the substrate binding pocket within ~4.5 angstroms from substrate are labeled and shown as ball-and-stick models with carbon atoms colored in green.

**Fig. 5:**
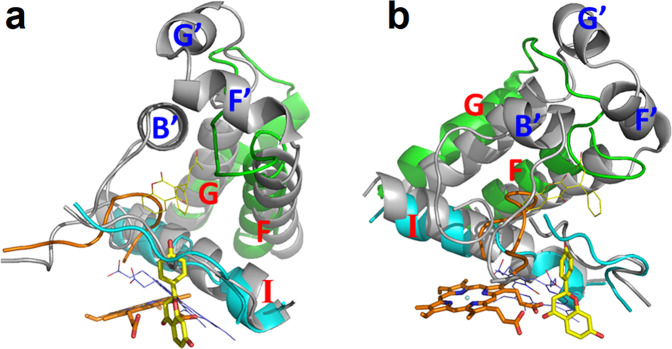
Conformation of the entrance to the 2-HIS active site. The conformation is shown in two different orientations with the plane of the heme ring perpendicular to the paper and the heme propionate groups pointing to the front (**a**) and with the heme propionate groups pointing to the right (**b**). The structure of 2-HIS is illustrated using different colors: the B-C loop (orange), helices F and G and F-G loop (green), helix I and strand β1-4 (cyan); and human P450 2C9 is in grey. The substrate and heme in 2-HIS are shown as thick bond models in yellow and orange, respectively, and the substrate and heme (thin bond model) in human P450 2C9 are in yellow and blue, respectively.

An observed structural feature of 2-HIS is a large pocket at the edge of the heme, close to its propionate groups (Fig. 4), and this is likely the substrate binding pocket. The location is similar to that of the substrate binding pockets in other reported P450 structures. Due to the heme being shifted away, the pocket becomes deep with its bottom on the loop before helix L (e.g., Phe437), and close to helices L (Ala455) and K (Phe363). The bottom of this pocket is ~6 Å deeper than that in other known P450 structures in which substrates locate near the surface of the heme. In the top of the pocket, the conformation of the substrate entrance regions is also quite different from that in other known P450 structures (Fig. 5). The B-C loop before helix C in 2-HIS is about eleven amino acids shorter than the corresponding helix B´ region in human P450 2C9. The F-G loop in 2-HIS is also about eight amino acids shorter than the corresponding region in human P450 2C5 which contains extra helices F´ and G´. These shorter sequences in 2-HIS result in a substantial shift of entrance down, ~10 Å, toward the new heme location.

Direct visualization of substrate binding by co-crystallization of 2-HIS with substrates and soaking of native crystals with substrates repeatedly proved unsuccessful. Due to the large shift of the substrate entrance region in the 2-HIS structure, a large cavity is formed on the molecular surface. Interestingly, the N-terminal region before helix A and strands β1-1 and β1-2 of another molecule of the dimer fit into this cavity (Supplementary Fig. 3b). The regions before and after helix A and residues in β2-2 are joined to the N-terminal transmembrane domain at Pro35 and likely interact with membrane, but are buried in the interface of the dimer. This may favor stable packing of molecules to facilitate crystallization, but also explains unsuccessful co-crystallization with substrates.

Molecular docking with substrate liquiritigenin showed that the C3 atom of substrate fits close to the heme propionate groups. The interactions between substrate and the enzyme are mainly hydrophobic with many hydrophobic residues (i.e., Leu113, Val 119, Phe363, Leu369, Val372, Val397, Phe437, Leu439, Leu440, and Leu497) in the binding pocket (Fig. 4). Phe363 may form a strong hydrophobic interaction with the aromatic A-ring of the substrate. Other hydrophobic residues (e.g., Leu369, Val372 and Leu439) may also form Van der Waals interactions with substrate. However, polar residues are also observed in the substrate binding pocket, Asn117 could interact with the substrate via a hydrogen bond, and Ser308 is close to both the heme and substrate. A mutational study showed that S308A had no detectable enzyme activity, indicating the key role of Ser308 in catalysis.

Asn117, Asp116, Leu113, Val119, Lys373, and Arg374 are close to the 4´-OH group on the B-ring of the substrate and likely control the ring migration. It has been reported that K375T mutant of licorice 2-HIS (CYP93C2) displayed the sole enzyme activity of producing 3-hydroxyflavanone without ring migration^27^, and the corresponding residue is Lys373 in *Medicago* 2-HIS. Therefore Lys373 plays a key role in the aryl migration reaction, possibly by interacting with the 4´-OH of the substrate. However, Lys373 also forms a salt-bridge with Asp116 in the B-C loop to help determine the conformation and location of this key structural feature. Mutation of Lys373 would disrupt the salt bridge with Asp116 and also the conformation of the B-C loop, and further abolish the 2-HIS ring migration activity.

### Implications for 2-HIS catalytic mechanism

In an early model of the 2-HIS catalytic mechanism^28^, it was proposed that the activated oxygen intermediate bound to the heme iron first abstracts the 3β-hydrogen from C3 of the flavanone substrate. However, based on the present 2-HIS structure, the substrate is not able to fit on top of the heme iron, being located in a large pocket at the edge of the heme and close to its propionate groups. The iron-bound oxygen in the activated oxygen intermediate is therefore too far away from the substrate to be inserted directly into the substrate through the proposed common P450 hydroxylation mechanism.

**Fig. 6:**
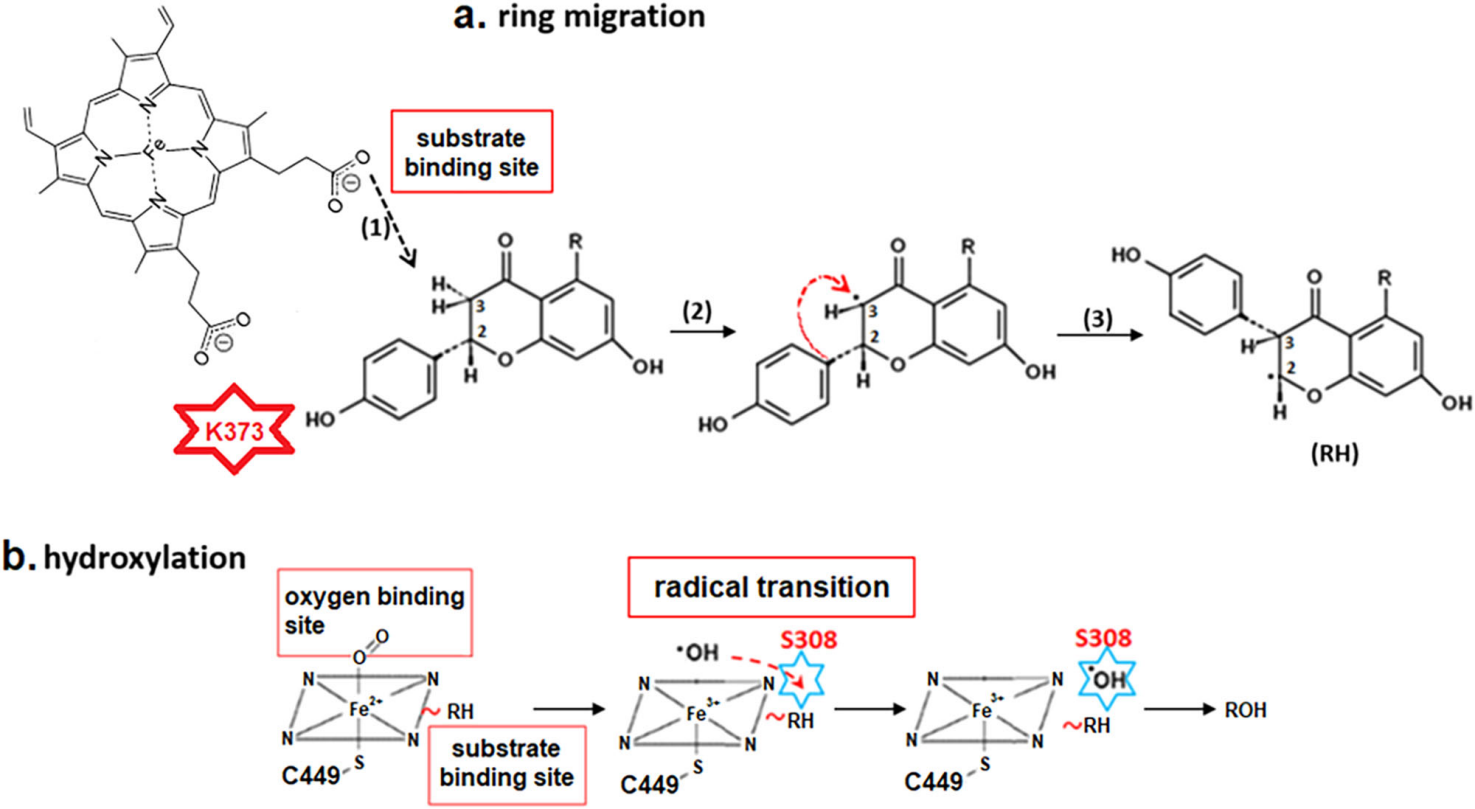
Proposed 2-HIS reaction mechanism. **a** Ring migration: Substrate binds near heme propionate groups (1); heme carboxylate abstracts 3β-hydrogen to deprotonate C3 (2); and B-ring moves from C2 to C3 (3). **b** Hydroxylation: The dioxygen is bound to the heme iron in the “oxygen binding site”; after the hydroxyl radical is generated, it would be transferred to the “hydroxyl radical transition site” near the heme propionate groups under Ser308, then finally transferred from the activated oxygen intermediate to the C2 atom after the migration of the phenyl B-ring from C2 to C3.

Similarly, the heme iron in the structures of peroxidases, e.g. horseradish peroxidase (HRP), is also not accessible and its substrates interact with the *δ-meso* heme edge^29^. HRP also functions as a monooxygenase, and the oxygen is introduced by reaction of a normal peroxidative product with oxygen or water. In the structure of 2-HIS, the site in the distal side of the heme iron is perfect for dioxygen binding. A hydroxyl radical, one type of reactive oxygen species (ROS) generated by microsomal P450 systems^30,31^, can also be formed in this “oxygen binding site”; then 2-HIS could possibly utilize a ferryl oxygen transfer mechanism^29^ to transfer hydroxyl radical to the “hydroxyl radical transition site” near the heme propionate groups under Ser308. The activated oxygen intermediate may interact with the C3 atom of substrate through the heme propionate groups to abstract the 3β-hydrogen, followed by transfer of the hydroxyl radical to the C2 atom of the substrate after the migration of the phenyl B-ring from C2 to C3 (Fig. 6).

**Fig. 7:**
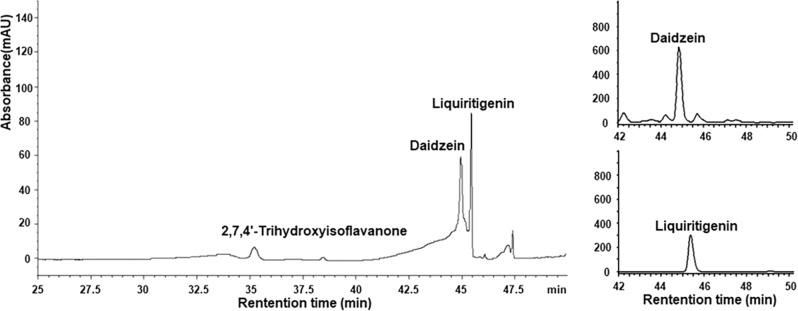
HPLC profiles of 2-HIS reaction products. 2-HIS enzyme was from dissolved crystals, and substrate liquiritigenin was converted to 2,7,4’-trihydroxyisoflavanone which was subsequently dehydrated to daidzein. The right panels are the HPLC profiles of authentic standards liquiritigenin and daidzein.

It is also possible that the heme and its surrounding protein regions, such as helix C, could undergo a conformational change and move toward the substrate binding pocket when 2-HIS binds to NADPH cytochrome P450 reductase and substrate. However, the substrate binding pocket then becomes too small and shallow and substrate would be exposed on the enzyme surface (Fig. 5) if the heme co-factor moved to the location characteristic of other known P450 structures. The reduced-CO difference spectrum was recorded with the 2-HIS protein and revealed a characteristic peak at 450 nm (Supplementary Fig. 5a), and the ligand-free 2-HIS exhibited a Soret absorption maximum in its UV-visible spectrum at 417 nm (Supplementary Fig. 5b), confirming that the 2-HIS protein was in a native state. Enzyme activity assays were also carried out with 2-HIS enzyme from dissolved crystals and showed that the enzyme was catalytically active (Fig. 7), indicating that the unique heme conformation in the crystals is not an artifact caused by denaturation.

In summary, the shortening of the B-C and F-G loops in 2-HIS results in a deep location for the substrate entrance region, and the substrate binding pocket is further moved down with the heme co-factor moved ~8 Å away. Together with the differences in helix I and the heme binding regions, these structural features of 2-HIS facilitate the ring-migration which is not a function of any other P450s with known structure.

### 2-HID structure

**Fig. 8:**
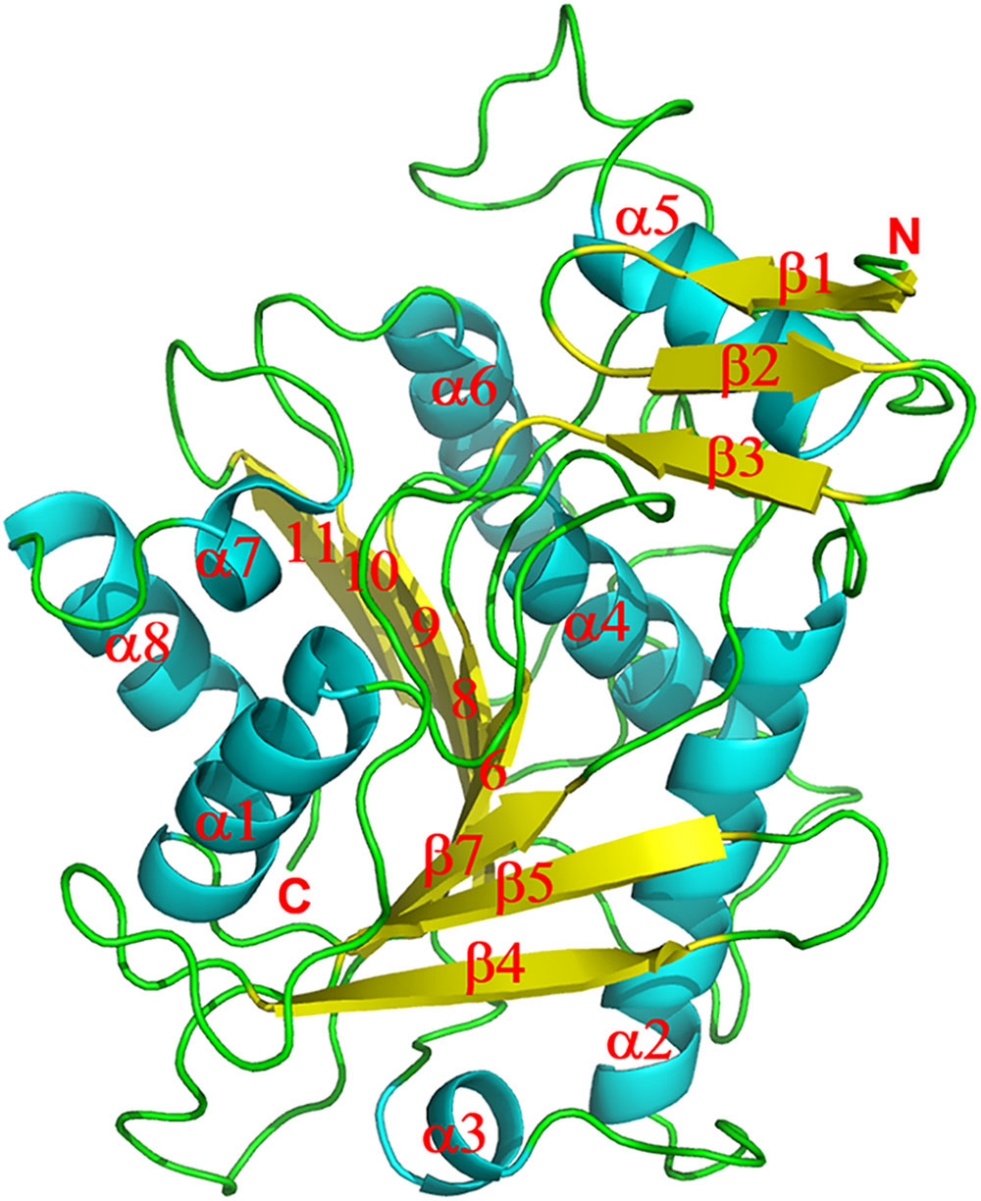
Ribbon diagram of the P. lobata 2-HID structure. The α helices, β strands and the N- and C-termini are labeled.

The crystal structure of kudzu 2-HID was determined at 2.4 Å resolution by the molecular replacement method (Table 2). The structure of 2-HID has a characteristic α/β-hydrolase fold, its core domain consists of a central eight-stranded β-sheet with three and four α helices packed on each side, and its N-terminal region contains a three-stranded β-sheet (Fig. 8 and Supplementary Fig. 6). 2-HID has been classified into a large carboxylesterase family although it is a dehydratase. Structural comparison through a DALI search showed that the 2-HID structure is most similar to that of the plant carboxylesterase AeCXE1 from *Actinidia eriantha*^32^ (Supplementary Figs. 6-7). Superimposing the structure of 2-HID onto that of AeCXE1 (PDB 2O7R) revealed strong structural similarity with a root-mean-square deviation (rmsd) of 2.6 Å for 290 Cα atoms and 34% sequence identity (Supplementary Figs. 6-7). The largest difference between these two enzymes was observed in the N-terminal region which presents a different conformation. The N-terminal region of AeCXE1 has only a two-stranded β-sheet which rotates ~40-60° compared with the corresponding region of 2-HID. Other differences were observed in a region before helix α5 (Supplementary Fig. 8).

**Table 2:** Summary of 2-HID data collection and refinement statistics.

Data statistics	Native	complex with *p* - nitrophenyl
Space group	*P*3_1_21	*P*3_1_21
Unit Cell	a = 102.3 Å	a = 102.8 Å
	b = 102.3 Å	b = 102.8 Å
	c = 70.2 Å	c = 70.3 Å
Resolution(Å)	2.4	2.3
Unique reflections	16673 (1693)	19522 (1912)
Completeness(%)	98 (100)	99.8 (100)
*R*_sym_ (%)*	8.3 (59.3)	5.4 (54.8)
I/σ(I)	14.8 (2.4)	42.2 (5.0)
*Refinement Statistics*		
R factor (%)	18.6	17.7
R_free_ (%)	24.4	20.0
Number of protein atoms	2455	2442
Number of water molecules	78	83
Average B-factors (Å^2^)	59.0	58.0
R.m.s.d. from ideal values		
Bond length (Å)	0.009	0.008
Bond angle (°)	1.274	1.127
Ramachandran plot		
Favored (%)	96	96
Allowed (%)	4	4
Outliers (%)	0	0

^*^R_sym_ = Σ_hkl_ | *I*- < *I* > | /Σ*I*, where *I* is the observed intensity and <*I* > is the average intensity from observations of symmetry-related reflections. A subset of the data (10%) was excluded from the refinement and used to calculate the free R value (R_free_). R factor = Σ||F_o_ | -|F_c_ | |/Σ|F_o_ | . Numbers in parentheses are for the highest resolution shell.

### 2-HID substrate binding pocket

**Fig. 9:**
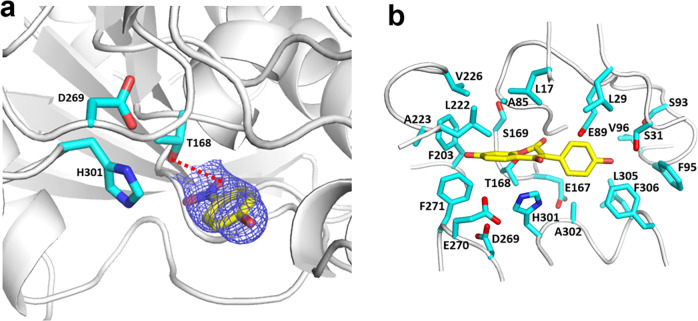
Structural basis for dual functionality of 2-HID from P. lobata. **a** 2Fo-Fc electron density map at 1.0 σ for *p*-nitrophenyl (NPO) and a catalytic triad, Thr168, Asp269 and His301 for carboxylesterase activity; **b** Active site of 2-HID docked with substrate 2,7,4´-trihydroxyisoflavanone close to the catalytic acid Glu89 and catalytic base His301. Key residues involved in substrate binding are shown as bond models and colored according to element: oxygen, red; nitrogen, blue; and carbon, cyan. NPO and 2,7,4’-trihydroxyisoflavanone are also shown as bond models with their carbon atoms in yellow.

Although co-crystallization of 2-HID with various substrates and analogs was explored, only a structure of 2-HID complexed with *p*-nitrophenol, a product of the carboxylesterase reaction, was obtained and well-defined electron density was observed for the ligand (Fig. 9a). The ligand is located in a pocket (Fig. 9a, b) formed by the loops β1-β2 (P16-L17) and β3-β4 (L29-S31) in the N-terminal region, helix α5 (L222-V226), loop β10-α4 (D269-F271), and helix α7 (H301-F306), including many hydrophobic residues (e.g., Leu17, Leu29, Ala85, Leu222, Ala223, Val226, Phe203, Phe271, and Phe305). In the bottom are Thr168 and Ser169 in helix α4 and a glycine-rich motif, Gly83-Gly84-Ala85, in loop β6-α1. Several charged residues are located in the pocket, including Glu89, Asp269, Glu270, and His301, which may play key functional roles. The location of this pocket is similar to that of the substrate-binding pocket identified in the structure of carboxylesterase AeCXE1^32^ and is also similar to the gibberellin (GA) binding pocket observed in the structure of gibberellin receptor GID1^33^. Compared with AeCXE1, the active site and substrate binding pocket of 2-HID are relatively open and large (Supplementary Fig. 8). In the structures of AeCXE1 and GID1, the loops before helix α5 are relatively long, with four more amino acids, and cover the top and form one side of the substrate binding pocket. In the 2-HID structure, this loop is away from the active site; this could be changed to a closed conformation when substrate binds to the enzyme.

The N-terminal region is also an important portion of the substrate binding pocket. In 2-HID, one end of the three-stranded β-sheet including Pro16, Leu17, Leu29, and Ser31 forms one large side of the substrate binding pocket. In AeCXE1, only two β strands were observed in the corresponding N-terminal region and are located in a different orientation (Supplementary Fig. 7), with the N-terminal end portion folding toward the active site and making the entrance smaller compared with the 2-HID structure. In GID1, two helices are present in the N-terminus before the two β strands and are extended to cover the entrance of the GA-binding site.

### The 2-HID active site and implications for dual functions and mechanism

The active site of AeCXE1 was identified in the substrate binding pocket with three catalytic triad residues Ser169, Asp276 and His306. In the structure of GID1, the corresponding location was identified as the GA binding site with two conserved carboxylesterase active residues Ser191 and Asp289, with the His replaced by Val319. Sequence and structural comparative studies with AeCXE1 showed that 2-HID possesses two corresponding catalytic triad residues Asp269 and His301, and the catalytic Ser is replaced by Thr168. These three residues may also form the catalytic triad for 2-HID carboxylesterase function. As observed in the structure of the complex of 2-HID with ligand, *p*-nitrophenol forms a hydrogen bond with Thr168 which is regarded as the nucleophilic residue for AeCXE1. Its ester hydrolysis mechanism would be similar to that of AeCXE1 and other carboxylesterases, and the nucleophilic hydroxyl of Thr168 in the catalytic triad acts as the catalyst. However, in the 2-HID structure, the CG2 atom of Thr168 is close to His301 and its OG1 atom points to another side and forms a hydrogen bond with *p*-nitrophenol. The orientation and conformation of His301 in 2-HID is also slightly different from those of His306 in AeCXE1. In addition, Ser169 in 2-HID is also close to *p*-nitrophenol and could interact with substrate, whereas the corresponding residue in AeCXE1 is Ala170.

The 2-HID mutants D269A, H301A and T168A had no or weak carboxylesterase activity on the *p*-nitrophenyl valerate, and the activity of mutant S169A was reduced to 52 % of that of wild-type enzyme (Table 3). Similarly, soybean 2-HID mutants D263N and H295A lost the carboxylesterase activity, and activity of mutants T164A and T164S was reduced to 1–3% of the wild type enzyme^8^. These studies suggest that Thr168, Asp269 and His301 may form a catalytic triad similar to that of AeCXE1, and Ser169 may also play a role in catalysis for carboxylesterase function.

**Table 3:** Dehydration and carboxylesterase activity of kudzu 2-HID enzyme and mutants.

	carboxylesterase activity	dehydration activity
	(%)	(%)
2-HID	100	100
E89A	23 ± 5.7	0
E89Q	50 ± 0.8	0
T168A	3 ± 0.5	1 ± 0.2
S169A	52 ± 1.1	0
D269A	0	3 ± 0.3
H301A	0	0

The relative activities to wild-type enzyme were obtained from identical incubations with equal amounts of protein, and the data are from 3 independent experiments.

It is likely that His301 acts as the catalytic base to abstract a hydrogen at C3 of the substrate and plays a similar catalytic role in the dehydration event. Molecular docking showed that Glu89 is close to the substrate, and its side chain can interact with the C2 hydroxyl of substrate (Fig. 9b and Supplementary Fig. 8). Glu89 is highly conserved in the 2-HID enzyme family, but Phe97 and Ser127 are present in the corresponding position in the structures of CXE1 and GID1, respectively. This Glu89 in 2-HID may act as an acid to protonate and abstract the hydroxyl to complete the elimination of water from the substrate. The H301A, E89A and E89Q mutations completely abolished the dehydration activity, but E89A and E89Q still had carboxylesterase activity, about 23% and 50% of wild-type enzyme activity, respectively (Table 3). Thus, both His301 and Glu89 play essential roles for the dehydratase function, and His301, but not Glu89, is also essential for the carboxylesterase function.

### Implications for 2-HID substrate specificity

Two types of 2-HID have been characterized: the enzyme from *G. echinata* is specific for the 4’-*O*-methylated 2-hydroxyisoflavanones and is designated as HIDM (i.e., methoxy type), and the soybean enzyme has broader specificity for both 4’-hydroxylated and -*O*-methylated substrates and is designated HIDH (i.e., hydroxy type)^8^.

Sequence comparison and structural analysis showed that amino acids forming the substrate binding pockets are highly conserved in *P. lobata* 2-HID, soybean HIDH and *G. echinata* HIDM. There are, however, some minor differences in the substrate binding pocket. *P. lobata* 2-HID has Ser31, Leu222, Ala302, Phe306, soybean GmHIDH has the same residues in the corresponding positions, but *G. echinata* GeHIDM has Gly34, Ser225, Cys305, and Tyr309.

Molecular docking showed that the 4´-hydroxyl of 2-hydroxyisoflavanone points near Ser31 and Phe306 in the single *P. lobata* 2-HID (Fig. 9b and Supplementary Fig. 8), which is similar to the soybean enzyme and should have broader specificity for forming both the 4´-hydroxylated and -*O*-methylated isoflavones found in this *P. lobata*^34^. In GeHIDM, Gly34 corresponds with Ser31 of *P. lobata* 2-HID. A substrate with a 4´-methoxy group would fit well into the large hydrophobic GeHIDM substrate binding pocket.

In summary, we describe the structural features of 2-HIS and 2-HID which together catalyze the entry reactions into the isoflavonoid pathway. 2-HIS possesses a currently unique structure that accounts for its performing both ring migration and hydroxylation, and 2-HID possesses dual enzymatic activities as a dehydratase and carboxylesterase and utilizes a new mechanism for the dehydration of isoflavones. In combination with our previous work on structural characterization of the two downstream enzymes isoflavone reductase^35^, vestitone reductase^36^, (iso)flavonoid glycosyltransferases UGT85H2^37^ and UGT78G1^38^, these structures provide a basis for the modification and engineering of the isoflavonoid biosynthetic pathway for formation of new isoflavone structures with enhanced bioactivities.

## Methods

### Cloning, expression, and protein purification

*M. truncatula* 2-HIS with its N-terminal transmembrane anchors replaced with the polypeptide MAKKTSSGR were cloned into pCWori+ vector with a four-histidine tag at the C-terminus. *E. coli* BL21(DE3) cells transformed with the plasmid were grown at 37 °C in LB medium containing 100 μg/ml ampicillin, 1 mM thiamine and 0.5 mM δ-aminolevulinic acid until A_600nm_ = 0.3–0.5. Cultures were induced with 1.0 mM isopropyl 1-thio-β-galactopyranoside (IPTG) and grown for 2 days at 30 °C. Cells were pelleted and resuspended in lysis buffer (50 mM Tris-HCl, pH 8.0, 500 mM NaCl, 10 mM imidazole, 10 mM β-mercaptoethanol). After lysis with an EmulsiFlex-C5 homogenizer (Avestin) and centrifugation at 12,000 rpm at 4 °C for 20 min, Ni^2+^-NTA agarose was added to the supernatant containing the target proteins. After incubation for 40–60 min, the mixture was transferred into a disposable column and washed extensively with lysis buffer. The His-tagged proteins were eluted with elution buffer (50 mM Tris-HCl pH 8.0, 500 mM NaCl, 250 mM imidazole, 10 mM β-mercaptoethanol). The protein was further purified on a Superdex-200 gel filtration column (Amersham Pharmacia Biotech) and concentrated to 5 mg/ml in 10 mM NaCl, 10 mM Tris-HCl pH 7.5, 5 mM β-mercaptoethanol.

Selenomethionine- (SeMet) substituted 2-HIS was prepared by expressing the recombinant protein in *E. coli* B834 (DE3) cells (Novagen) grown in M9 minimal medium supplemented with SeMet according to the reported method with minor modifications^39^. The procedure for expression and purification was similar to the procedure described above for the native protein.

2-HID from kudzu was cloned into vector pDEST17. The resulting plasmid was transformed into *E. coli* strain BL21 (DE3) (Novagen). *E. coli* cells were grown at 37 °C in LB medium containing 50 μg/ml kanamycin until A_600nm_ = 0.6–0.8. After induction with 0.25 mM IPTG, the cultures were grown overnight at 16 °C. Cells were harvested by centrifugation at 5,000 rpm for 30 min and stored at −80 °C until purification. Cell pellets was resuspended in lysis buffer (50 mM Tris-HCl, pH 8.0, 500 mM NaCl, 10 mM imidazole, 20 mM β-mercaptoethanol). After lysis with an EmulsiFlex-C5 homogenizer (Avestin) and centrifugation at 12,000 rpm at 4 °C for 20 min., Ni^2+^-NTA agarose was added to the supernatant containing the target proteins. After incubation for 40–60 min., the mixture was transferred into a disposable column and washed extensively with the lysis buffer (about 50 column volumes). The His-tagged 2-HID proteins were eluted with elution buffer (50 mM Tris-HCl pH 8.0, 500 mM NaCl, 250 mM imidazole, 20 mM β-mercaptoethanol). The proteins were further purified with Resource Q and Superdex-200 gel filtration columns (Amersham Pharmacia Biotech) and concentrated to 20 mg/ml in 10 mM NaCl, 10 mM Tris-HCl pH 7.5, 5 mM β-mercaptoethanol.

### Mutagenesis and enzyme assay

Site-directed mutants of 2-HIS and 2-HID were constructed using the QuikChange strategy (Stratagene). The mutant proteins were expressed and purified using the procedures for native proteins.

The 2-HIS activities of wild-type and mutant enzymes and dissolved 2-HIS crystals were determined mainly according to a reported method^27,40^. A 300 µl reaction mixture included 0.1 M K_2_HPO_4_, pH 8.0, 0.4 M sucrose, 0.5 mM glutathione, 3 mM NADPH, 160 µM liquiritigenin or naringenin, and approximately 4 mg microsomes containing *Arabidopsis thaliana* NADPH-P450 reductase ATR1 prepared from yeast WAT11 cells transformed with pYeDP60 vector^41^. The reaction was started by added 25 µg protein or crystals and was extracted with ethyl acetate after incubation at 16 °C for 12 h and acid treatment with 10% (v/v) HCl at room temperature for 1 h. The extract was evaporated to dryness under vacuum and HPLC analysis of product carried out with a Waters Spherisorb 5 μ ODS2 C18 reverse phase column (250 × 4.6 mm) on an Agilent HP1100 HPLC equipped with an autosampler, a quaternary pump, and a diode array detector. Both substrate liquiritigenin and product daidzein were verified by HPLC analysis of authentic standards and comparison of absorption spectra and retention times.

The dehydratase activity of 2-HID was determined according to a reported method^8^ with some modifications. A 300 µl reaction mixture included 0.1 M K_2_HPO_4_, pH 8.0, 0.4 M sucrose, 0.5 mM glutathione, 3 mM NADPH, 300 µM liquiritigenin, and approximately 5 mg microsomes containing *A. thaliana* NADPH-P450 reductase AtATR1 prepared from yeast WAT11 cells transformed with *M. truncatula* 2-HIS pYeDP60 vector. The reaction was terminated by extraction with ethyl acetate after incubation at 30 °C for 2 h. The extract was concentrated under reduced pressure until to dry, and the reaction products resuspended in reaction buffer (100 mM phosphate pH 8.0) at various concentrations as 2-HID substrates. The reaction was started by addition of 0.1 µg purified wild-type or mutant 2-HID proteins and incubated in a final volume of 100 µl with the reaction buffer. The reaction was carried out at 30 °C for 30 min and then terminated with the addition of 100 µl methanol. The solution was centrifuged at 14,000 *g* for 20 min and 100 µl of solution was injected for HPLC analysis as above.

2-HID carboxylesterase activity was measured against *p*-nitrophenyl valerate. A reaction mixture comprising 50 mM PBS buffer, pH 7.1 and 1 mM substrate containing 1% acetonitrile as solvent in a final volume of 100 µl. The reaction was initiated by adding approximately 40 ng - 10 µg 2-HID wild-type or mutant proteins. The reaction was carried out at 40 °C for 1 h and then terminated with the addition of 100 µl methanol. After centrifugation at 12,000 *g* for 20 min, the supernatant (100 µl) was subjected to HPLC analysis as above.

### Crystallization and data collection

Crystals of 2-HIS were grown from hanging drops by the vapor diffusion method. 2-HIS protein at a concentration of 5 mg/ml was mixed with an equal volume of reservoir solution containing 200 mM (NH_4_)_2_SO_4_, 200 mM ammonium tartrate dibasic, 50 mM imidazole, 1% FOS-choline-8, 30% (w/v) PEG3350, and 0.1 M K_2_HPO_4_/NaH_2_PO_4_ (pH 6.8). The mixture was equilibrated over the reservoir solution at 20 °C. Two different forms of crystals were obtained. Se-Met derivative crystals were grown under similar conditions with 1% FOS-choline-8 replaced by 1% NDSB-221.

Prior to data collection, the crystals were flash-cooled to −180 °C. Data from a native 2-HIS protein crystal were measured to 2.0 Å with an ADSC Quantum 315 CCD detector at the SBC 19ID beamline of the Advanced Photon Source. The 2-HIS crystal belonged to space group P2_1_ (a = 49.8 Å, b = 73.2 Å, c = 148.4, β = 93.3°). There were two molecules per crystallographic asymmetric unit with 47.7% solvent content and a V_M_ of 2.4 Å3/Da. A 3.0 Å MAD data set from a SeMet derivative crystal, using two wavelengths, was collected at the Stanford Synchrotron Radiation Lightsource (SSRL) beamline BL9-2 using a MAR325 CCD detector. The crystal belonged to a different space group P2_1_2_1_2_1_ (a = 73.8 Å, b = 96.0 Å, c = 154.6 Å).

Crystals of *P. lobata* 2-HID were grown from hanging drops using the vapor diffusion method. Two μl of a 20 mg/ml solution of protein was mixed with 2 μl of reservoir solution (18% PEG3350, 0.1 M HEPES pH7.5). The mixture was equilibrated over the reservoir solution at 20 °C. Crystals grew over 3–5 days to the dimensions of 0.3 × 0.2 ×0.1 mm. The crystals were flash frozen at −180 °C. Data from a crystal of 2-HID were measured to 2.4 Å resolution with a conventional X-ray source (RU-H3R) and Raxis IV + + image plate detector. The crystal belonged to space group P3_1_21 (a = 101.9 Å, b = 101.9 Å, c = 69.7 Å) with 42% solvent content. There was one molecule in the crystallographic asymmetric unit.

Crystals of 2-HID complexed with *p*-nitrophenol were obtained by co-crystallization. 2-HID protein at a concentration of 20 mg/ml was mixed with *p*-nitrophenol and incubated at 4 °C for 2 h, then co-crystallization was performed using the same condition identified for the wild type enzyme. Data from the crystal of 2-HID complexed with *p*-nitrophenol were measured to 2.3 Å with an ADSC Quantum 315 CCD detector at the SBC 19ID beamline of the Advanced Photon Source. The crystal belonged to the same space group P3_1_21 as the native 2-HID crystal.

All data sets were indexed, integrated, and scaled using the HKL2000 software package^42^.

### Structure determination and refinement

The structure of 2-HIS was determined using the multiwavelength anomalous dispersion (MAD) method. The MAD data (40-3.0 Å) from a crystal in P2_1_ space group were analyzed with Auto-Rickshaw^43^, yielding an overall figure of merit of 0.44, and an initial model was automatically built. This model was used as a template to determine native 2-HIS structure in P2_1_2_1_2_1_ space group using the molecular replacement method with the program PHASER^44^. Interactive model building and crystallographic refinement were carried out using the programs COOT^45^, CNS^46^, and Phenix Refine^47^, respectively. A bulk solvent correction was applied. B factors were refined individually for native protein structure at 2.0 Å. Water molecules were added with Arp/wArp^48^ and checked manually for inclusion. In the models, the first six (in molecule A) or seven (in molecule B) amino acid residues in the N-terminus and last nine residues in the C-terminus were not observed in molecules A and B, and several regions (A97-107, A284-A288, A422-430, A435-437, B95-107, B227-229, B284-287, and B421-B430) were disordered.

The structure of *P. lobata* 2-HID was solved by molecular replacement using the program PHASER^44^ and a plant carboxylesterase AeCXE1 structure (PDB ID: 2O7R) as a search model. Interactive model building and crystallographic refinement were carried out using programs COOT^45^, CNS^46^ and Phenix^47^, respectively. A bulk solvent correction was applied. B factors were refined individually. Water molecules were added with Arp/wArp and checked manually for inclusion. The first eight amino acid residues in the N-terminus were not observed.

The program PROCHECK^49^ was used to check the model. All backbone ϕ-ψ torsion angles are within allowed regions of the Ramachandran plot.

### Molecular docking

The automated docking program GOLD^50^ was used to dock substrate into the 2-HIS and 2-HID active sites. Default genetic algorithm parameters for controlling the operation of the docking process were used. All docking calculations were restricted to the predicted binding pocket by defining the active site with heme co-factor (for 2-HIS) or residue His301 (for 2-HID). For 2-HID, the location of ligand in the structure bound with *p*-nitrophenol was used as reference. GOLDscore was used to identify the lowest energy docking results. The hydrogen bonds and van der Waals contacts between ligands and enzyme were analyzed to identify the optimal binding mode. Minor manual adjustments of the GOLD solution were made using the program COOT.

### Statistics and reproducibility

The 2-HID enzyme assays were performed in triplicates, and the activity data are presented as mean±standard error of the mean. The protein purification experiments for both 2-HIS and 2-HID were repeated over three times, which presented almost identical sample features. For the structures reported in the paper, X-ray diffraction data sets were collected with 2–5 crystals, and the best data sets with high diffraction quality were used for structural determination and refinement. The data collection and refinement statistics are summarized in Tables 1 and 2.

### Reporting summary

Further information on research design is available in the Nature Portfolio Reporting Summary linked to this article.

## Supplementary information

Supplementary Information

Reporting Summary

## Data Availability

Atomic coordinates and structure factors for the reported crystal structure has been deposited with the Protein Data Bank under the accession codes 8E83 for 2-HIS and 8EA1 and 8EA2 for 2-HID.
