# Supplementary material for: The protein conformational basis of isoflavone biosynthesis
Source: Commun Biol. 2022 Nov 15;5:1249. doi: 10.1038/s42003-022-04222-x (PMC9663428; doi:10.1038/s42003-022-04222-x)
Supplement: Supplementary file 1 — Supplementary Information [file 42003_2022_4222_MOESM1_ESM.pdf]

Supplementary Information

The protein conformational basis of isoflavone biosynthesis

Xiaoqiang Wang[1,4✉], Haiyun Pan[2,4], Someswar Sagurthi[3], Vincent Paris[1], Chunliu Zhuo[1], and Richard A. Dixon[1]

[1] BioDiscovery Institute and Department of Biological Sciences, University of North Texas, Denton, Texas 76203-5017, USA

[2] Conagen, Inc., Bedford, MA 01730, USA

[3] Department of Genetics & Biotechnology, Osmania University, Hyderabad, India 500007

[4] These authors contributed equally: Xiaoqiang Wang, Haiyun Pan.

✉ email: Xiaoqiang.Wang@unt.edu

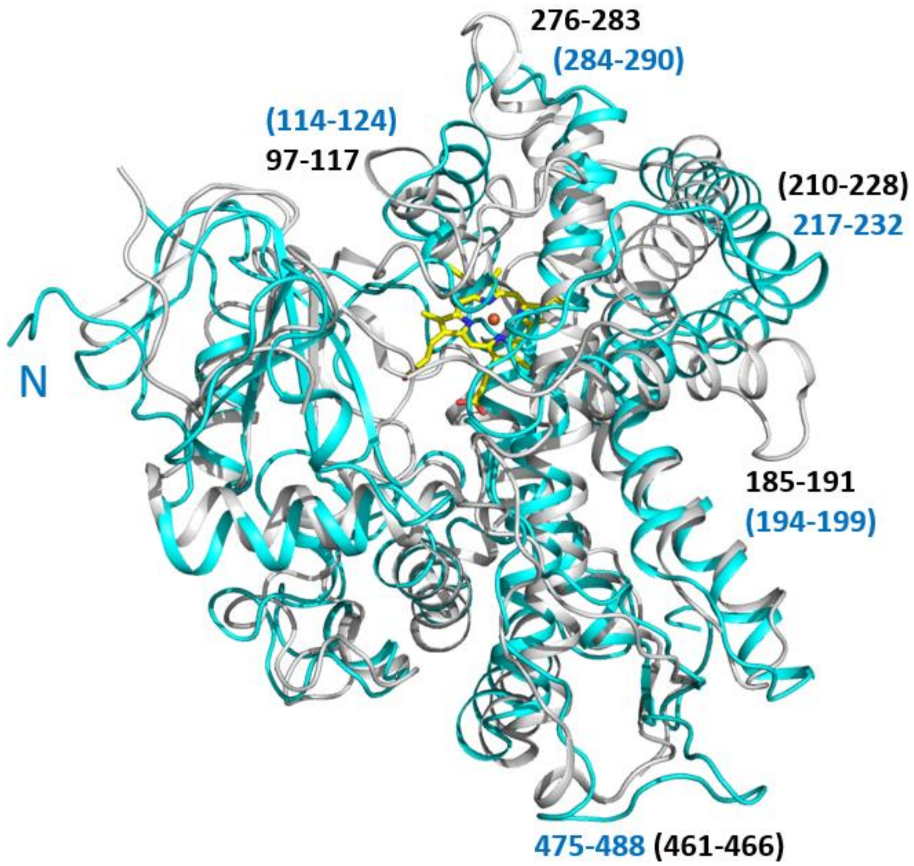

Supplementary Figure 1. Comparison of structures of 2-HIS (cyan) and CYP 2C9 (grey).

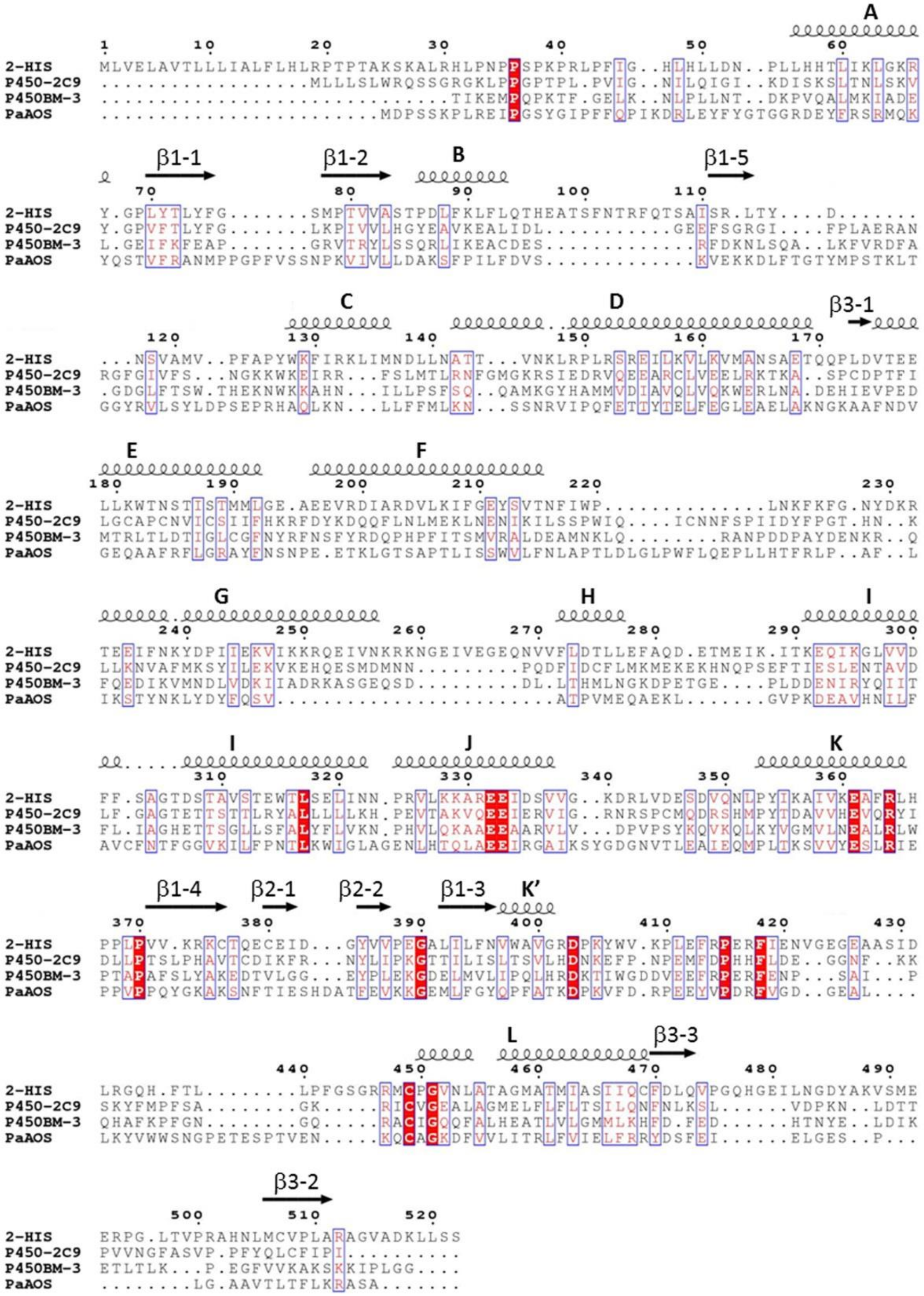

Supplementary Figure 2. Sequence alignment of *Medicago* 2-HIS, human P450 2C9, *Bacillus megaterium* P450BM-3 and the plant soluble P450 allene oxide synthase (AOS). The secondary

structure elements observed in the 2-HIS structure are shown above the alignment. Conserved residues are highlighted.

a

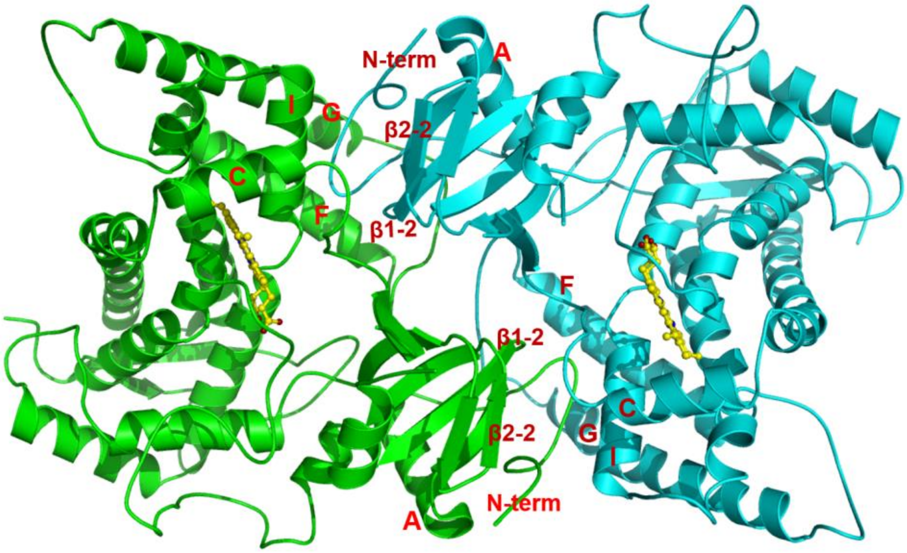

b

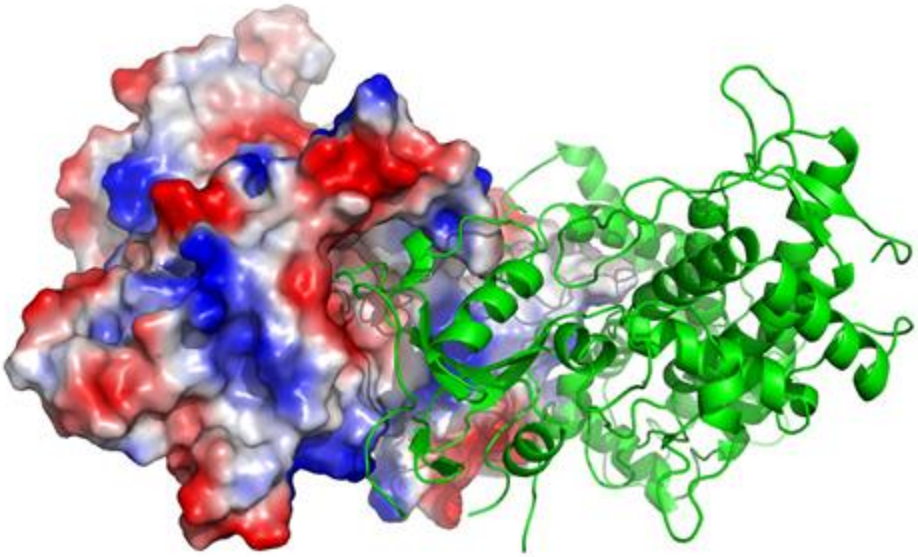

Supplementary Figure 3. 2-HIS dimer in crystal lattice. **a.** Ribbon diagram with one monomer shown in green and the other in cyan. **b.** Molecular surface of one monomer showing the cavity and substrate entrance fitted by the other monomer.

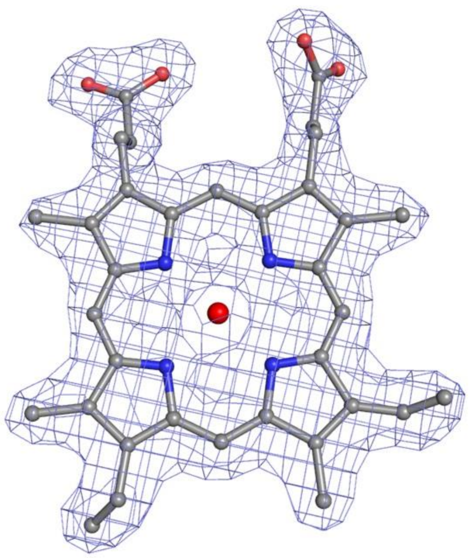

Supplementary Figure 4. 2Fo-Fc electron density map of heme contoured at 1.0 σ.

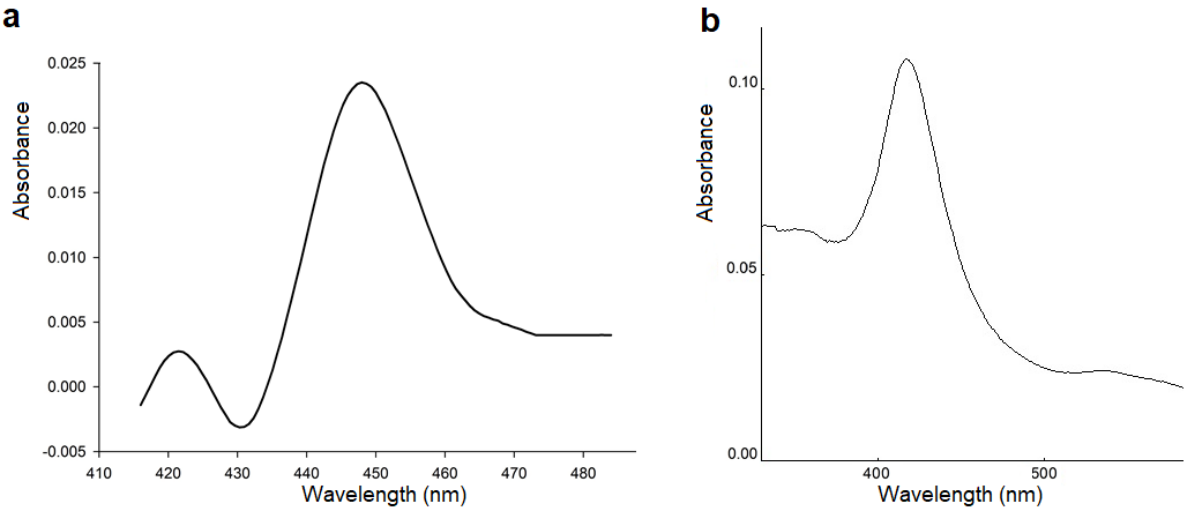

Supplementary Figure 5. (a) CO difference spectrum of 2-HIS. Two ml purified 2-HIS enzyme in Tris-HCl buffer (50 mM, pH 8.0) was divided in two cuvettes (sample and reference) and 2 mg of solid $Na_2S_2O_4$ was added to each cuvette. Baseline absorption was determined using a split-beam spectrophotometer. CO was then bubbled through the sample cuvette for a few seconds, and the difference spectrum was recorded. The reduced-CO difference spectrum of the 2-HIS enzyme had a characteristic peak at 450 nm. **(b)** UV-vis spectrum of 2-HIS in the absence of the substrate, and the ligand-free 2-HIS exhibited a Soret absorption maximum in its UV-visible spectrum at 417nm.

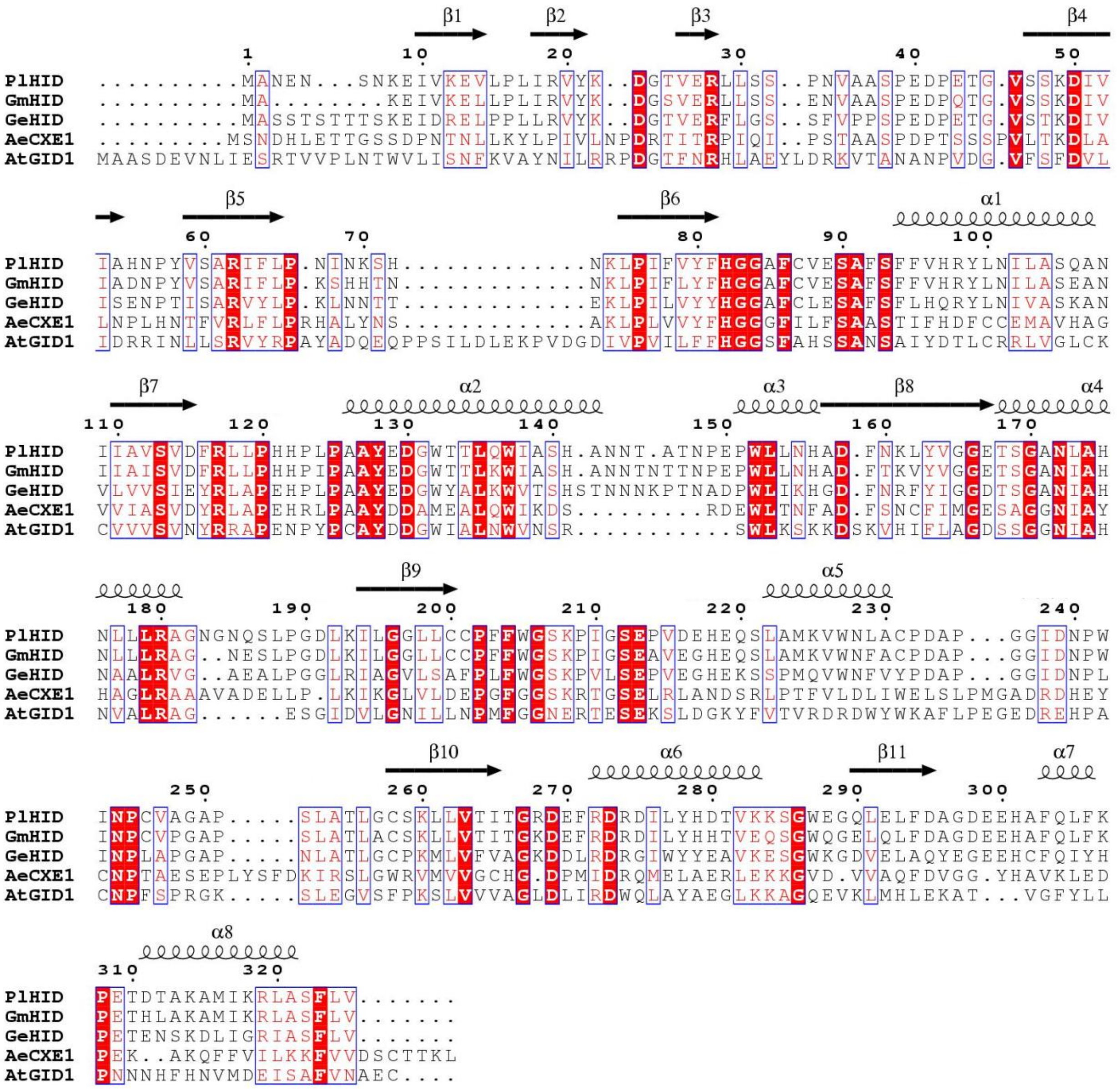

Supplementary Figure 6. Sequence alignment of kudzu 2-HID, soybean GmHID, *Glycyrrhiza echinata* GeHID, AeCXE1and *A. thaliana* gibberellin receptor AtGID1.

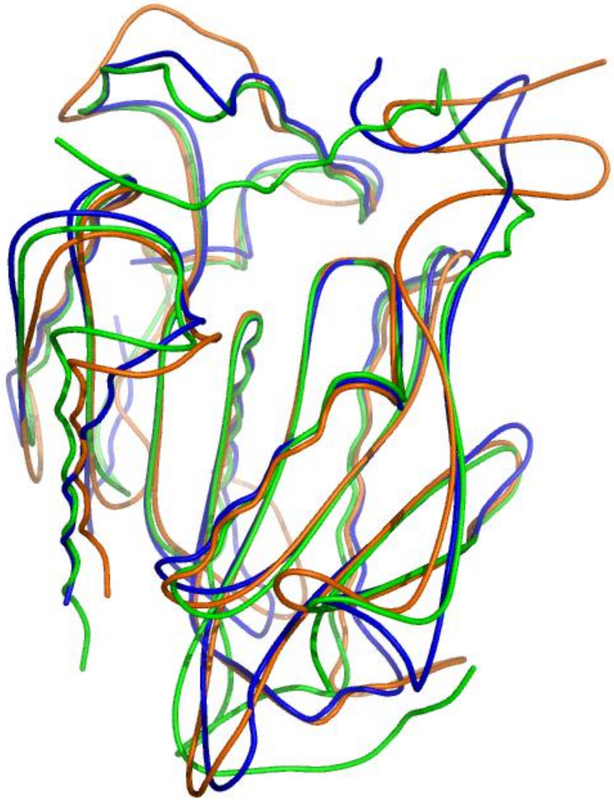

Supplementary Figure 7. Structural comparison of 2-HID (orange), CXE1 (blue) and GID1 (green).

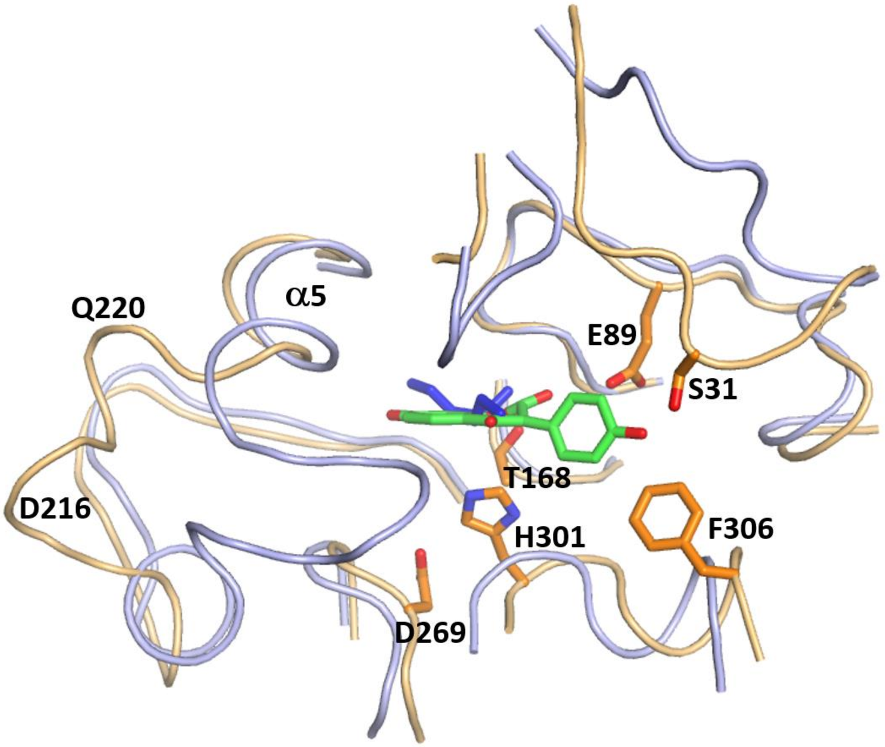

Supplementary Figure 8. Substrate binding pockets of 2-HID (light orange) docked with substrate 2,7,4'-trihydroxyisoflavanone (color codes: carbon, green; oxygen, red) and CXE1 (light blue) bound with inhibitor paraoxon (blue). The catalytic triad residues Thr168, His301, and Asp269 of 2-HID are shown as bond models and colored according to element: oxygen, red; nitrogen, blue; and carbon, orange.
